# Rising Trends in Pediatric Type 2 Diabetes: A Comprehensive Bibliometric Analysis of Global Research (1998-2023)

**DOI:** 10.31729/jnma.v63i2091.9223

**Published:** 2025-11-30

**Authors:** Raju Vaishya, Anoop Misra, Brij Mohan Gupta, Ghouse Modin Naseesab Mamdapur, Abhishek Vaish

**Affiliations:** 1 Indraprastha Apollo Hospitals, New Delhi, India; 2 Fortis Centre of Excellence for Diabetes, Metabolic Diseases & Endocrinology Hospital for Diabetes and Allied Sciences, New Delhi, India; 3 Formerly with Centre of Excellence for Diabetes, Metabolic Diseases & Endocrinology, New Delhi, India; 4 Yenepoya (Deemed to be) University, Mangalore, Karnataka, India; 5 Indraprastha Apollo Hospitals, New Delhi, India

**Keywords:** *diabetes mellitus*, *pediatrics*, *bibliometrics*, *childhood obesity*, *insulin resistance*, *diabetes research*

## Abstract

Pediatric Type 2 Diabetes is a growing global concern, driven by rising childhood obesity and lifestyle factors. The study aimed to analyze global publication trends, key contributors, and collaborative networks in Pediatric Type 2 Diabetes research. A bibliometric analysis of 1,555 Scopus-indexed publications was conducted. Study types, citation metrics, and collaboration networks were evaluated. There was 20.02% annual growth (5 papers in 1998 to 99 in 2023). The USA (42%) led globally, followed by the UK, Canada, and Germany; Finland had the highest citation impact. Original research (70.6%) dominated, mainly on clinical and epidemiological themes. P. Zeitler was the most prolific author, while G. Imperatore’s works were the most cited. Strong collaborations existed between the USA and the UK, with the University of Colorado Anschutz Medical Campus as the leading institution. Pediatric Type 2 Diabetes research shows rapid expansion but limited contributions from India and South Asia, highlighting a need for region-specific studies.

## INTRODUCTION

Type 1 diabetes (T1D) is the most common form of diabetes in children,^1^ yet type 2 diabetes (T2D) now accounts for nearly one-third of new pediatric cases.^2^ Initially regarded as adult-onset, T2D has increasingly been reported in obese youth since the 1960s-70s.^3-7^ Its prevalence has risen globally, paralleling the childhood obesity epidemic and presenting with more aggressive progression and earlier complications than T1D.^8-9^ Pediatric T2D (PT2D) affects diverse populations and is projected to quadruple in U.S. youth by 2050, particularly among minorities.^10-11^ Recent global data confirm rising incidence and disability burden, especially in low- and middle-income regions.^12-15^ However, bibliometric analyses of PT2D research remain scarce despite multiple studies on T1D and T2D.^16-23^

## METHODS

A bibliometric analysis of PT2D literature was conducted using the Scopus database on March 10, 2024, recognized for its extensive scholarly coverage.^22^ The publication date of literature is from January 1, 1998 to December 31, 2023. The search strategy in Scopus' "Advance Search" option was:

(TITLE (type 2 diabetes* OR diabetes, AND type 2) AND TITLE (child* OR adoles* OR pediat* OR paediat* OR juven* OR kids OR newborn* OR infant*)) AND ( EXCLUDE ( PUBYEAR , 2024 ) OR EXCLUDE ( PUBYEAR , 1998 ) OR EXCLUDE ( PUBYEAR , 1997 ) OR EXCLUDE ( PUBYEAR , 1996 ) OR EXCLUDE ( PUBYEAR , 1995 ) OR EXCLUDE ( PUBYEAR , 1994 ) OR EXCLUDE ( PUBYEAR , 1983 ) OR EXCLUDE ( PUBYEAR, 1982)

The 1555 retrieved publications were analyzed using MS Excel and VOSviewer.^24^ VOSviewer was used to generate network visualization maps, with Total Link Strength (TLS) indicating international research collaboration. The analysis included evaluating annual publication productivity, journal productivity and impact, and the productivity and impact of countries, organizations, and authors using publication counts, average citations per publication (CPP), and relative citation index (RCI). Collaboration networks were visualized using TLS. The top articles were defined as those with greater CPP and RCI than the average of the study. The top 99 most cited articles were summarized, and keyword co-occurrence, thematic maps, and trend-topic analysis were performed to gain insights into PT2D research. Bibliometric indicators were used to assess productivity (publication count), impact (citations), and connections (structural indicators), with the RCI comparing citation rates to the global average.^25^ This study also analyzed publications related to PT2D with a focus on various demographics and regions.

## Results

### OVERALL PICTURE

A bibliometric analysis of 1555 papers on PT2D published between 1998 and 2023 revealed a significant annual growth rate of20.02%, increasing from 5 publications in 1998 to 99 in 2023, with peak outputs in 2021 (104) and 2022 (121). The cumulative number of publications grew by 64.45% from 1998-2010 (588) to 1998-2023 (967). These publications received a total of45758 citations, averaging 39.43 CPP, which showed a declining trend from 45.92 CPP to 19.40 CPP, likely due to the shorter citation window for more recent publications (Table 1).

Original research articles constituted the majority (70.60%), followed by reviews (15.43%). Research focused predominantly on clinical studies (49.9%), epidemiology and risk factors (29.07%), and pathophysiology (13.18%). Controlled studies (53.11%) were the most common research design. External funding supported 29.71% of the publications, contributing 45.11% of the total citations, with the National Institutes of Health being the top funding agency. International collaboration was involved in 39.54% of the publications, accounting for 51.79% of the total citations, with the USA being the leading collaborating country.

### PRODUCTIVE AND IMPACTFUL COUNTRIES

Bibliometric analysis reveals a global engagement in PT2D research, with 84 countries contributing. The top 15 countries produced 90.35% of the publications (1405 papers) and accounted for over 100% of the citations (54947). The USA (n=655), UK (n=122), Canada (n=104), and Germany (n=104) surpassed the average productivity (93.67 papers). Finland (CPP=77.87, RCI=2.65), UK (CPP=55.89, RCI=1.90), Israel (CPP=46.13, RCI=1.57), India (CPP=44.69, RCI=1.52), Sweden (CPP=43.38, RCI=1.47), Denmark (CPP=42.0, RCI=1.43), USA (CPP=41.01, RCI=1.39), and Australia (CPP=39.8, RCI=1.35) exceeded the average citation impact (CPP=39.11, RCI=1.33). International collaboration among the top 10 countries averaged 34.23% (Table 2). Notably, there is a concerning gap in research output from India and the broader South Asian context.

**Figure 1:**
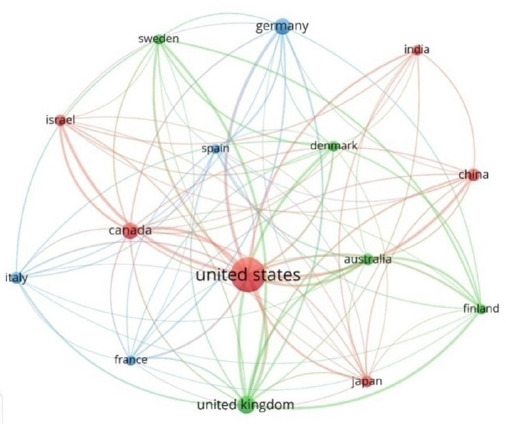
Collaborative Network Map of Top 15 Countries with 29 or more publications in Pediatric Type 2 Diabetes Research.

The USA and UK showed the highest collaborative linkages (n=152 each) among the top 15 most productive countries (Figure 1), which formed three distinct collaborative clusters. The strongest bilateral collaboration was between the USA and the UK (n=20).

### PRODUCTIVE AND IMPACTFUL AUTHORS

Analysis of 6071 authors contributing to 1555 publications on PT2D identified the top 30 authors, who produced 470 papers (30.23% of the total), garnering 26679 citations (58.30%). Predominantly from the USA (n=22), these authors exhibited varying productivity and impact, with 13 exceeding the average of 15.67 papers and 11 surpassing the average citation impact of 56.76 CPP and 1.93 RCI (profiles of the top six in each category are in Table 3).

**Table 1:** Annual Growth of Publications on Pediatric Type 2 Diabetes during 1998-2023

Year	TP	TC	CPP	Year	TP	TC	CPP
1998	5	373	74.60	2013	59	1872	31.73
1999	16	1574	98.38	2014	53	2702	50.98
2000	26	4163	160.12	2015	62	1541	24.85
2001	27	1419	52.56	2016	81	1734	21.41
2002	45	1756	39.02	2017	59	1756	29.76
2003	44	3161	71.84	2018	86	2350	27.33
2004	66	1777	26.92	2019	64	897	14.02
2005	74	2768	37.41	2020	69	1102	15.97
2006	67	1874	27.97	2021	104	1205	11.59
2007	64	2185	34.14	2022	121	990	8.18
2008	56	2125	37.95	2023	99	196	1.98
2009	47	1453	30.91	1998-2010	588	27002	45.92
2010	51	2374	46.55	2011-2023	967	18756	19.40
2011	41	1007	24.56	1998-2023	1555	45758	29.43
2012	69	1404	20.35				

TP = Total papers; TC = Total citations; CPP = Citations per paper

**Table 2:** The top 15 countries/regions dealing with Pediatric Type 2 diabetes research

S. No	Country	TP	TC	CPP	RCI	%TP	ICP	%ICP	TLS	TLS-INST	TLS-WN	TLS-INS-WN
1	United States of America	655	26863	41.01	1.39	42.12	155	23.66	152	44	138	14
2	United Kingdom	122	6818	55.89	1.90	7.85	66	54.10	152	44	95	14
3	Canada	104	2570	24.71	0.84	6.69	39	37.50	56	32	47	13
4	Germany	104	3008	28.92	0.98	6.69	39	37.50	86	31	44	12
5	China	56	1038	18.54	0.63	3.60	16	28.57	25	18	28	7
6	Japan	47	1371	29.17	0.99	3.02	7	14.89	30	21	23	14
7	Australia	46	1831	39.80	1.35	2.96	26	56.52	57	25	44	11
8	Italy	45	1469	32.64	1.11	2.89	15	33.33	40	20	23	11
9	Israel	39	1799	46.13	1.57	2.51	23	58.97	46	18	34	10
10	Denmark	34	1428	42.00	1.43	2.19	21	61.76	87	39	41	12
11	France	32	1186	37.06	1.26	2.06	10	31.25	53	34	28	12
12	Sweden	32	1388	43.38	1.47	2.06	23	71.88	60	20	37	10
13	Finland	31	2414	77.87	2.65	1.99	22	70.97	65	25	44	11
14	India	29	1296	44.69	1.52	1.86	7	24.14	25	15	17	8
15	Spain	29	468	16.14	0.55	1.86	12	41.38	62	29	23	12
	**A total of 15 countries**	1405	54947	39.11	1.33	90.35	481	34.23	996	415	666	171
	Global total	1555	45758	29.43	1.00	100.00						
	Share of top 15 countries in global total	90.35										

TP=Total papers; TC=Total citations; CPP=Citations per paper; RCI=Relative citation index; ICP=International collaborative papers; TLS=Total link strength

**Table 3:** Top six most productive and most impactful authors contributing to research related pediatric type 2 research

S. No.	Name of the author	Affiliation of the author	TP	TC	CPP	RCI	%TP	ICP	%ICP	TLS	TLS-INST
**Top six most productive authors**											
1	P. Zeitler	University of Colorado School of Medicine, USA	27	2068	76.59	2.60	1.74	13	48.15	187	157
2	T. Urakami	Nihon University School of Medicine, Japan	24	528	22.00	0.75	1.54	2	8.33	100	60
3	O. Pinhas-Hamiel	Chaim Sheba Medical Center Israel	22	1395	63.41	2.15	1.41	16	72.73	160	62
4	D. Dabelea	Colorado School of Public Health, USA	21	2624	124.95	4.24	1.35	2	9.52	214	128
5	W.V. Tamborlane	Yale School of Medicine, USA	21	782	37.24	1.27	1.35	7	33.33	240	124
6	R.W. Holl	Universitat Ulm, Germany	20	678	33.90	1.51	1.29	11	52.38	162	98
**Total six most impactful authors**											
1	Imperatore, G.	National Center for Chronic Disease Prevention and Health Promotion, USA	11	2402	218.36	7.43	0.71	1	4.76	136	89
2	Arslanian, S.	UPMC Children’s Hospital of Pittsburgh, USA	11	2066	187.82	6.39	0.71	4	19.05	298	160
3	D. Dabelea	Colorado School of Public Health, USA	21	2624	124.95	4.24	1.35	2	9.52	214	128
4	Goran, M.I.	University of Southern California, USA	15	1298	86.53	2.94	0.96	2	9.52	72	40
5	Pihoker, C.	University of Washington, USA	13	1053	81.00	2.76	0.84	2	9.52	181	120
6	Klingensmith, G.J.	Barbara Davis Center for Diabetes, USA	14	1076	76.86	2.61	0.90	4	19.05	180	128

(TP=Total papers; TC=Total citations; CPP=Citations per paper; RCI=Relative citation index; ICP=International collaborative papers; TLS=Total link strength)

The share of internationally co-authored papers (ICPs) among the top 24 authors ranged from 0.0% to 72.73%. Collaboration among the top 30 authors, measured by TLS, ranged from 56 to 425, with F. Bacha (TLS=425) showing the strongest collaborative links. The co-authorship network of these top 30 authors (Supplementary Figure 1) forms seven distinct clusters, illustrating collaboration patterns and highlighting key contributors in the field, with author node size proportional to their contribution.

### PRODUCTIVE AND IMPACTFUL ORGANIZATIONS

Analysis of 1555 publications on PT2D revealed the involvement of 615 organizations, with the top 30 contributing 20-63 papers each (total 942 papers, >100% of citations, 60.58% of publications). Twenty-six of these top organizations were in the USA, and one each in Canada, Germany, Israel, and Japan. Among them, 11 exceeded the average productivity (31.40 papers), and 10 surpassed the average citation impact (52.42 CPP.1. 78 RCI). Table 4 presents the top six organizations in terms of both productivity and citation impact.

These leading 30 organizations showed strong collaboration, with TLS ranging from 43 to 512. The highest TLS was observed for the University of Colorado Anschutz Medical Campus (n=512), Cincinnati Children's Hospital Medical Center (n=434), and the University of Colorado School of Medicine (n=413). The strongest organizational collaboration link (n=31) was between Cincinnati Children's Hospital Medical Center and the University of Cincinnati.

A collaborative network map of these top 30 organizations (Supplementary Figure 2) shows 2985 links with a TLS of 6751, indicating strong interconnections. These organizations are grouped into six clusters, revealing distinct collaborative networks.

### PUBLISHING JOURNALS

Analysis of journal distribution (Supplementary Table 1) within the 1555 publications on PT2D reveals that *Pediatric Diabetes* is the most productive journal, with 114 publications, followed by *Diabetes Care* (88) and *Journal of Pediatric Endocrinology and Metabolism* (49). In terms of citation impact (CPP), *The Lancet* exhibits the highest CPP at 155.29, followed by *JAMA* (150.33) and *Pediatrics* (138.06). Regarding Journal Impact Factor (IF), *The Lancet* again leads with an IF of 98.4, followed by the *New England Journal of Medicine* (96.2) and *JAMA* (63.1), indicating that while *Pediatric Diabetes* publishes the most articles in this field, journals with broader scope and higher impact factors tend to receive more citations.

### SIGNIFICANT KEYWORDS

**Figure 2:**
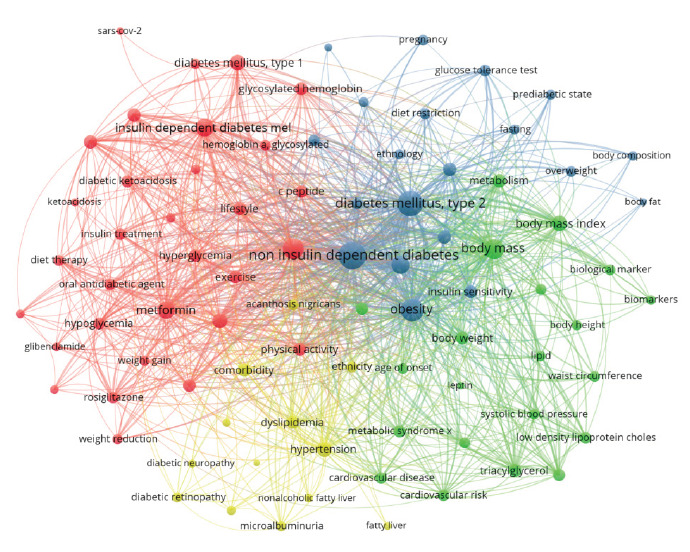
The clustering and co-occurrence of 88 terms keywords of publications related to pediatric type 2 diabetes (Software VOSviewer; n =>19)

The co-occurrence network analysis of 7251 keywords from 1555 publications identified research directions and hotspots in PT2D, revealing a structure organized into four distinct clusters (Figure 2). These 1449 links with a TLS of 3380 indicate strong keyword inter-connectedness. Cluster 1 (31 keywords, Red) centres on insulin (1277 occurrences, 128 links, 680 TLS), type 1 diabetes, and related treatments. Cluster 2 (21 keywords, Green) focuses on body mass (484), body mass index (198), and metabolic factors. Cluster 3 (20 keywords, Blue) highlights noninsulin-dependent diabetes mellitus (1278), type 2 diabetes (939), obesity (665), and insulin resistance (347). Finally, Cluster 4 (11 keywords, Yellow) includes hypertension (172), comorbidity (124), and dyslipidemia (115). These clusters, derived from 73 important keywords (appearing ≥4 times out of 330), represent key thematic areas and emerging trends in PT2D research.

### TOP-CITED PUBLICATIONS

Out of 1555 publications, 99 (3.67%) were highly cited (HCPs), receiving 100-1103 citations (average 240.33 CPP, total 23793). Among these, 40 received ≥200 citations, 21 ≥300, 13 ≥400, and 9 ≥500. Funding supported 45 HCPs, and 26 involved international collaboration, while 55 had national collaboration and 18 had no collaboration.

Over 50 countries contributed to HCPs, with the USA leading (n=57), followed by the UK (n=21), Germany (n=8), Finland (n=7), Canada and Japan (n=5 each). For countries with ≥3 papers, Israel had the highest CPP (295.25), followed by Finland (291.0), India (272.33), and the USA (259.42). Over 100 organizations were involved, with the University of Colorado Anschutz Medical Campus (n=11), Cincinnati Children's Hospital Medical Center and UPMC Children’s Hospital of Pittsburgh (n=10 each) having the most contributions. Among organizations with ≥4 papers, the University of Washington showed the highest CPP (417.4), followed by the National Institute of Diabetes and Digestive and Kidney Diseases (387.0) and Cincinnati Children's Hospital Medical Center (352.5). The 300 authors from these organizations were led by S. Arslanian (n=9, UPMC), P. Zeitler (n=7, Colorado), and G. Imperatore (n=6, CDC). Among authors with ≥3 papers, D. Dabelea had the highest CPP (452.6), followed by G. Imperatore (384.17) and C. Pihoker (368.0).

Ninety-nine HCPs were published in 27 journals. *Diabetes Care* had the most HCPs (n=18), followed by *Journal of the Endocrine Society* and *Pediatrics* (n=8). By citation impact, the *Journal of Pediatrics* had the highest CPP (530), followed by *JAMA* (434.2) and *The Lancet* (294.33). Journal Impact Factors ranged from 1.30 to 98.4.

**Table 4:** Top six most productive and most impactful institutions contributing to research related to pediatric type 2 diabetes research

S. No	Affiliation of the institute	TP	TC	CPP	RCI	%TP	ICP	%ICP	TLS	TLS-INST
Top six most productive organizations										
1	University of Colorado Anschutz Medical Campus, USA	63	2916	46.29	1.57	4.05	14	22.22	512	152
2	Cincinnati Children's Hospital Medical Center, USA	63	4798	76.16	2.59	4.05	10	15.87	434	150
3	University of Colorado School of Medicine, USA	60	2665	44.42	1.51	3.86	19	30.16	413	157
4	Yale School of Medicin, USA	45	1190	26.44	0.90	2.89	10	15.87	269	117
5	University of Colorado Department of Pediatric, USA	43	1949	45.33	1.54	2.77	13	20.63	395	143
6	University of Manitoba, Canada	40	1069	26.73	0.91	2.57	8	12.70	179	93
Top six most impactful organizations										
1	National Institute of Diabetes and Digestive and Kidney Diseases, USA	26	2878	110.69	3.76	1.67	4	6.35	187	95
2	University of Washington School of Medicine, USA	22	2389	108.59	3.69	1.41	6	9.52	183	82
3	University of Washington, USA	25	2620	104.8	3.56	1.61	9	14.29	238	108
4	Colorado School of Public Health, USA	27	2351	87.07	2.96	1.74	2	3.17	222	72
5	UPMC Children’s Hospital of Pittsburgh, USA	30	2434	81.13	2.76	1.93	5	7.94	158	89
6	Keck School of Medicine of USC, USA	28	2199	78.54	2.67	1.8	1	1.59	108	60

TP=Total papers; TC=Total citations; CPP=Citations per paper; RCI=Relative citation index; ICP=International collaborative papers; TLS=Total link strength

## DISCUSSION

This bibliometric study analyzed 1555 publications on PT2D from 1998-2023, revealing a significant increase in research interest with an average annual growth rate of 20.02%. The research involved 6071 authors across 76 countries and generated 45758 citations (average CPP of 39.43), with original research comprising the majority (70.60%). The USA was the leading contributor in publication volume (42.12%, 655 papers), while Finland demonstrated the highest citation impact among top countries (CPP of 77.87). The University of Colorado Anschutz Medical Campus and Cincinnati Children's Hospital Medical Center were the most productive institutions, and P. Zeitler was the most prolific author.

The study highlighted strong international collaboration between the USA and the UK. Academic journals were the primary dissemination avenue, with *Pediatric Diabetes* publishing the most articles and *The Lancet* exhibiting the highest citation impact (CPP of 155.29). Keyword analysis identified four key thematic areas within PT2D research. The analysis underscores a growing global focus on PT2D, with the USA dominating research output. At the same time, other nations and specific journals demonstrate notable influence within the field.

This bibliometric analysis did not explicitly focus on comparative studies within PT2D research. However, the high prevalence of publications related to "clinical studies" (49.9% share) and "treatment outcome" (83 publications) suggests a significant interest in comparing different interventions and management strategies in this population. Furthermore, the keyword analysis revealed terms related to "insulin resistance," "obesity," and "complications," which are often central to comparative studies examining different phenotypes and disease trajectories within PT2D. Wu et al. (2022) in a systematic review found that the top 10 treatments of PT2D were saxagliptin+metformin, liraglutide+metformin, liraglutide, dapagliflozin, exenatide-2 mcg, sitagliptin+metformin, linagliptin-5 mg, linagliptin-1 mg, metformin, and exenatide-5/10 mcg.^26^ Bjornstad et al. (2022) confirmed that PT2D is becoming more common globally, affecting all racial and ethnic groups, with indigenous people and people of colour being disproportionately impacted. This rise is driven by factors like increasing childhood obesity, exposure to diabetes in the womb, inactive lifestyles, and systemic racism. Furthermore, they found PT2D to be more severe than in adults, with greater IR and faster decline of insulin-producing cells, leading to earlier development of complications. The limited availability of approved medications for PT2D makes treatment challenging.^2^ Effective interventions and strategies to improve adherence are crucial for managing this condition and preventing long-term complications in young people. Reinehr (2013) provided a comprehensive review comparing the characteristics and management of T2D in children and adults.^27^

The increasing research on "epidemiology and risk factors" often involves comparative analyses across different ethnic groups and geographical locations to understand the varying prevalence and determinants of PT2D.^28-30^ While Europe has lower overall rates, countries like Germany and the UK have reported significant increases. Asia is also experiencing a rise, with threefold increases in Hong Kong and substantial annual increases in China, where urban youth are at higher risk.^30^ A significant increase in PT2D prevalence in China was reported from a modeling study, with a projected increase of 26.6% per year in youth aged 10-19. Specifically, the prevalence is expected to rise from 1.77 per 1000 individuals in 20172019 to even higher levels in the future.^31^ PT2D is increasing dramatically with the high-speed urbanization of China, with its prevalence varying from 1.64/100,000 to 15.16/100,000 based on the geography and economy. Monogenic diabetes used to be underestimated in China and now more cases are emerging.^32^

The burden of PT2D is on the rise globally, as well as in India.^33,34^ A recent systematic review of cohort studies revealed a wide range in the prevalence of PT2D across South Asia, from 0.1% to 28.3%. Specifically, prevalence in India ranged from 0.4% to 26.8%, while other South Asian countries showed a range of 0.1% to 28.3%, and migrant South Asians exhibited a prevalence between 4.1% and 18.1%.^35^ Although comprehensive national data for India is still developing, registry data suggests that about one in four diabetes cases in those under 25 is T2D, with a likely increasing trend linked to urbanization and rising obesity.^36^ In a systematic review by Wu et al. (2021), estimated its incidence in India at 397 per 100,000 ranking second in the world.^37^ In a study by Kumar et al. the prevalence of pre-diabetes/T2D was 12.3% and 8.4% among Indian adolescent boys and girls, respectively with obesity/overweight being an important predictor of disease.^38^ Nanditha et al. (2024) reported over a 10-year period, T2D prevalence significantly increased in Southern India for both younger (4.5% to 7.8%) and older individuals (28.4% to 34%). Notably, younger individuals also showed a more pronounced increase in obesity and dyslipidemia rates. In terms of incidence, T2D increased by 120% in younger individuals (0.5% to 1.1%) and by 150% in older individuals (2% to 5%) during the study.^39^

Published research on PT2D is noticeably lacking in India and South Asia, despite rising incidence linked to distinct factors like childhood obesity and socio-economic conditions. This bibliometric analysis highlights an urgent need for increased, tailored research in this region to inform effective public health interventions and policies. Collaborative efforts are crucial to address this gap and the growing prevalence of PT2D in South Asian youth.^40^,^41^

The rising obesity epidemic significantly drives PT2D through IR and P-cell dysfunction, often with a decade-long delay. Early life factors like in-utero over-nutrition and postnatal exposures (e.g., shorter breastfeeding, pollutants, unhealthy home environments) increase risk. Social determinants of health, including income, education, housing, food access, and healthcare, are crucial upstream factors contributing to a disproportionate burden of obesity and T2D in youth from lower socioeconomic backgrounds and marginalized populations facing poverty and structural racism. Addressing these risks requires tailored interventions for high-risk groups and community-wide changes promoting healthy lifestyles through improved resources and engagement.^42^

Study’s limitations: This study, while providing a broad overview of PT2D research, is limited by its reliance on the Scopus database, potentially missing relevant publications in other databases like Web of Science (WoS) or PubMed. However, combining bibliometric data from Scopus and WoS presents challenges due to differing data structures, indexing practices, and citation tracking, leading to data heterogeneity and duplicate records. Resolving these inconsistencies is essential for accurate analysis but requires significant effort in standardization and merging. Bibliometric analyses, including citation counts, offer an imperfect measure of research impact influenced by factors beyond a study's true significance. The keyword analysis offers a general thematic view, and a more detailed qualitative analysis could reveal finer research nuances. Finally, the study's findings reflect a snapshot up to 2023, and research trends are subject to change.

Future directions: Future research on PT2D should expand research in high-prevalence, low-resource areas; conduct longitudinal bibliometric studies; integrate bibliometric data for comprehensive impact assessment; and explore interdisciplinary collaborations. A key focus should be characterizing the unique spectrum of PT2D using clustering approaches (genetics, environment, social factors, clinical features) to improve understanding, prevention, diagnosis, and treatment. Understanding the intergenerational cycle of obesity and T2D, especially maternal diabetes impact, is vital for early prevention. A two-pronged prevention approach is needed: longitudinal studies to identify high-risk youth for targeted interventions, and collaborative efforts across sectors to implement healthy policies, accessible programs, and ensure screening and referral for equitable prevention and treatment.^42^

## CONCLUSION

This bibliometric analysis highlights a significant increase in publications on pediatric type 2 diabetes (PT2D) over the past two decades, reflecting a growing recognition of the condition’s prevalence and severity among youth. The annual growth rate of 20.02% indicates a need for ongoing research to address this rising health concern, particularly given its association with childhood obesity. Key contributors to the literature, including leading countries and organizations, were identified, emphasizing the collaborative nature of current research efforts. Furthermore, analyzing frequently cited studies and keyword trends offers insights into critical areas of focus within PT2D research. These findings highlight the importance of developing targeted interventions and policies to effectively combat the increasing incidence of PT2D in children and adolescents globally.
